# Household

**Published:** 1890-01

**Authors:** 


### HOUSEHOLD.

Chicken Pie.—Boil chickens until tender, line the sides of a pan with a rich biscuit dough, put in part of the chicken, season with salt, pepper and butter; lay on a few strips of the dough, add the rest of the chicken, season as before; season the liquor that chickens were boiled in, add part to pie, cover with a crust, with a hole in the center the size of a teacup, so as to add the gravy as is needed. Bake one hour in a moderate oven.

Mince Pie.—As most people can make the crust, I will only give directions for preparing the mince meat. Two bowls chopped apples, one of chopped meat, with one-fourth pound suet; grated rind and juice of one lemon, two teacups of molasses, one large teaspoonful each of cinnamon and cloves, one nutmeg, one pound raisins, half pound currants, one-fourth pound citrons cut fine, one quart cranberries, one quart cider: sugar and salt to taste.

Baked Beans.—One quart beans soaked over night; in morning put in fresh water and parboil, change water and boil until soft; put in a closely-covered earthern jar; season with pepper; add one-half cup molasses (New Orleans) and one-half pound of salt pork. Bake in a slow oven twenty-four hours, filling up with hot water when dry. These are the old-fashioned Boston Baked Beans.

Graham Bread.—With one quart warm water and one cup yeast, make a stiff batter with graham flour. Let rise, and add a half cup of brown sugar and one teaspoon of soda; mold into loaves with wheat flour; let rise and bake in a slow oven.

To Mix Table Mustard.—Get good mustard. Two or three hours before you want to use, mix it with lukewarm water; stir till about the consistency of thick cream; cover up until wanted for the table—then use vinegar to thin it. If this rule is strictly adhered to, the mustard will never turn black, nor will the vinegar rise to the top.

Batter Cakes.—One cup of meal, one cup of sweet milk, one cup of butter-milk, two eggs, tablespoonful of butter, half a teaspoonful each of salt and soda; bake on a hot griddle.

Coffee Cake.—One cup butter, two of sugar, one of treacle, five cups of flour, sifted, three eggs, one cup of strong, cold coffee, half a pound of raisins, stoned and floured; half a pound of currants, quarter of citron, chopped or sliced fine; one teaspoonful of powdered cinnamon, one of cloves, half a nutmeg, grated; one teaspoonful of soda, good measure. Good cake bakers always cream butter and sugar thoroughly, sift flour, and beat eggs separately very light, adding the whites last.

Cloths dipped in very hot water, wrung out and instantly applied to the seat of pain, will be frequently of very great service. In some instances it adds to the efficacy to employ substances having medical properties as well. In every process of fomentation, there should be two flannels, each three yards long, with the ends sewed together, and one flannel should be got ready while the other is on.

